# Propofol-mediated circ_0000735 downregulation restrains tumor growth by decreasing integrin-β1 expression in non-small cell lung cancer

**DOI:** 10.1515/med-2022-0539

**Published:** 2023-01-31

**Authors:** Lihui Zhang, Yunli Gao, Yue Li, Xinying Li, Haixia Gong

**Affiliations:** Department of Anesthesiology, Hulunbeier Municipal People’s Hospital (Hulunbuir Hospital Affiliated to Suzhou University Hulunbuir), Inner Mongolia, China; Department of Anesthesiology, Maanshan People’s Hospital, Maanshan, Anhui, China; Department of Anesthesiology, First Affiliated Hospital of Nanchang University, No. 17 Yongwaizhengjie Street, Donghu District, Nanchang, Jiangxi Province, 330006 China

**Keywords:** propofol, NSCLC, circ_0000735, ITGB1

## Abstract

Propofol, an intravenous anesthetic agent, exerts an anti-tumor peculiarity in multifarious tumors. Circular RNA hsa_circ_0000735 (circ_0000735) is involved in non-small cell lung cancer (NSCLC) progression. The purpose of this study is to investigate whether propofol can curb NSCLC progression via regulating circ_0000735 expression. Cell viability, proliferation, apoptosis, and invasion were detected using 3-(4,5-dimethylthiazol-2-yl)-2,5-diphenyltetrazolium bromide, 5-ethynyl-2′-deoxyuridine, flow cytometry, and transwell assays. Evaluation of protein levels was performed using western blotting or immunohistochemistry. Detection of circ_0000735 in tissue samples and cells was carried out using a real-time quantitative polymerase chain reaction. The molecular mechanisms associated with circ_0000735 were predicted by bioinformatics analysis and verified by dual-luciferase reporter assays. The relationship between propofol and circ_0000735 *in vivo* was verified by xenograft models. The results showed that circ_0000735 was overexpressed in NSCLC samples and cells. Propofol treatment overtly decreased circ_0000735 expression in NSCLC cells and repressed NSCLC cell viability, proliferation, invasion, and facilitated NSCLC cell apoptosis, but these effects mediated by propofol were counteracted by circ_0000735 overexpression. Circ_0000735 functioned as a miR-153-3p sponge and regulated integrin-β1 (ITGB1) expression via adsorbing miR-153-3p. ITGB1 overexpression reversed circ_0000735 silencing-mediated effects on NSCLC cell viability, proliferation, invasion, and apoptosis. In conclusion, propofol restrained NSCLC growth by downregulating circ_0000735, which functioned as a miR-153-3p sponge and regulated ITGB1 expression via adsorbing miR-153-3p. This study provides evidence to support that propofol curbs NSCLC progression by regulating circRNA expression.

## 1 Introduction

Currently, surgery is still the main treatment for cancer. A series of studies uncover that the management of anesthetics during surgery is related to the prognosis of cancer [1,2]. Retrospective clinical studies manifest that the prognosis of cancer surgery with intravenous anesthesia is better than that with inhalation anesthesia [3,4]. Propofol (2,6-diisopropylphenol), an intravenous anesthetic agent, is the most extensively used for induction and maintenance of anesthesia [5]. Recently, many studies have uncovered that propofol plays an anti-tumor peculiarity in multifarious tumors [6]. Also, propofol anesthesia is implicated in better survival in stage I non-small cell lung cancer (NSCLC) patients who underwent radical surgery [7]. However, the mechanisms by which propofol regulates NSCLC progression are unclear.

Circular RNAs (circRNAs), endogenous biomolecules, show differential expression among different species, developmental stages, and pathologies. They are characterized by a covalently closed loop structure produced by a special type of alternative splicing [8]. Most circRNAs have known functions to sequester proteins or microRNAs (miRs), translate to produce polypeptides, regulate transcription, and interfere with splicing [9]. Also, the deregulation of circRNAs is associated with the tumorigenesis of many cancers [10]. Recently, several studies have revealed that circRNAs participate in the repressive effect of propofol on tumor growth. For instance, propofol restrained tumorigenesis through downregulation of circ_PVT1 in gastric cancer [11], circ_TADA2A and circ_ERBB2 in lung cancer [12,13], and circ_VPS13C in ovarian cancer [14]. In contrast, propofol postponed tumor progression via upregulation of circ_0026344 in colorectal cancer [15]. CircRNA hsa_circ_0000735 (circ_0000735), located on chr17: 3802927-3808661, is generated from the P2RX1 gene. A previous study manifested that circ_0000735 exerted a tumor-promoting activity in NSCLC [16]. However, whether propofol can restrain progression by changing circ_0000735 expression is unclear.

Accordingly, the purpose of the research was to check whether propofol can curb NSCLC progression via regulating circ_0000735 expression.

## 2 Materials and methods

### 2.1 Surgical specimens

Thirty-two NSCLC patients were enrolled in the research after obtaining written informed consent. Lung tumors and paired non-tumor adjacent samples were collected during surgery at Hulunbeier Municipal People’s Hospital. The patients did not receive any preoperative treatment.


**Ethics approval and consent to participate:** Written informed consent was obtained from patients with approval by the Institutional Review Board in Hulunbeier Municipal People’s Hospital.

### 2.2 Cell culture

Human bronchial epithelial-like cells (HBE) (Procell, Wuhan, China), NSCLC cell lines H1299 and A549 (Procell), as well as HEK-293T cells (Procell) were cultured in Roswell Park Memorial Institute-1640 Medium, Ham’s F-12K, or Dulbecco’s Modified Eagle Medium supplemented with 10% FBS (Procell) and 1% penicillin/streptomycin (Procell) under the appropriate conditions (5% carbon dioxide and 37°C).

### 2.3 Propofol treatment

Dissolution of propofol (Sigma, St Louis, MO, USA) was performed using 10% intralipid (Astra-Zeneca, London, UK), followed by diluting with serum-free medium to a stock concentration of 0.4 mg/mL. For propofol treatment, the cells were cultured in the complete medium containing diverse doses of propofol (5, 10, and 15 μg/mL).

### 2.4 Plasmid construction and oligonucleotides

Full-length sequences of circ_0000735 and integrin-β1 (ITGB1) were, respectively, cloned into the empty pCD5-ciR vector (Geneseed, Guangzhou, China) and pcDNA vector (Thermo Fisher, Waltham, MA, USA) to establish pCD5-ciR-circ_0000735 (circ_0000735) and pcDNA-ITGB1 (ITGB1) plasmids. All oligonucleotides were synthesized by Sangon Biotech Co., Ltd (Shanghai, China), including a siRNA targeting circ_0000735 (si-circ_0000735), miR-153-3p inhibitor (anti-miR-153-3p), miR-153-3p mimic (miR-153-3p), and their negative controls si-NC, anti-miR-NC, and miR-NC. Transfection of the cells with oligonucleotides and/or plasmids was executed with Lipofectamine 3000 (Thermo Fisher). HEK-293T cells were transfected with the recombinant pLKO.1 vector (Addgene, Cambridge, MA, USA) carrying sh-circ_0000735 or sh-NC (1 μg) along with psPAX2 packaging plasmid (750 ng) and pMD2.G envelope plasmid (250 ng). Lentivirus particles from HEK-293T cells were collected and then used to infect A549 cells under polybrene (8 µg/mL, Sigma).

### 2.5 Detection of cell viability

After transfection, the cells were cultured in the complete medium containing diverse doses of propofol for 48 h. Following this, 10 μL 3-(4,5-dimethylthiazol-2-yl)-2,5-diphenyltetrazolium bromide (MTT) solution (Roche, Basel, Switzerland) was added. Then, 100 μL of dimethyl sulfoxide was used to dissolve the purple crystals. The optical density was evaluated using a microplate reader VarioSkan Flash (Thermo Scientific).

### 2.6 5-Ethynyl-2′-deoxyuridine (EdU) assay

Detection of cell proliferation was executed using an EdU Detection Kit (Ribobio, Guangzhou, China) according to the manufacturer’s protocol. Staining of the nucleus was performed with the 4’,6-diamidino-2-phenylindole solution (Thermo Fisher), and the proportion of EdU-positive cells was observed using fluorescence microscopy (Olympus, Tokyo, Japan).

### 2.7 Flow cytometry assay

Assessment of cell apoptosis was carried out using the Annexin V-FITC Apoptosis Detection Kit (Sigma). Briefly, the cells were washed and re-suspended in binding buffer. Next, the cells were incubated in the dark with Annexin V-FITC solution and propidium iodide solution, followed by flow cytometry analysis with an LSRII Fortessa flow cytometer (BD Biosciences, San Jose, CA, USA). Apoptotic rate was the sum of the early and late apoptotic rates.

### 2.8 Transwell invasion assay

Invasion capacity was evaluated using the CHEMICON Cell Invasion Assay Kit (ECM550, Sigma). In short, about 5 × 10^5^ cells were seeded on the top of the invasion chamber after 24 h of transfection. The cells were stained with 0.1% crystal violet (Sigma) after another 24 h of incubation. The number of invading cells was counted using an inverted microscope (Olympus, Tokyo, Japan).

### 2.9 Western blotting

Tissue specimens and cultured cells were lysed in ice-cold radio immunoprecipitation assay buffer solution. After centrifugation (10,000*g*, 10 min), the supernatants were collected, followed by quantifying with the BCA Protein Assay Kit (Beyotime). Proteins in equal concentration were electrophoresed in polyacrylamide gels and transferred onto nitrocellulose membranes (Thermo Fisher). Incubation with antibodies recognizing MMP9 (ab137867, 1:1,000, Abcam, Cambridge, MA, USA), Cleaved-caspase3 (ab32042, 1:500, Abcam), ITGB1 (ab134179, 1:1,000, Abcam), and GAPDH (ab128915, 1:10,000, Abcam) was carried for 16 h at 4°C after blocking with 5% milk. Membranes were then incubated with a secondary antibody (ab205718, 1:10,000, Abcam). Bands were detected using a chemiluminescence system (Thermo Fisher), followed by quantification using the ImageJ software (v1.8.0; NIH).

### 2.10 RNA isolation, RNase R digestion, complementary DNA synthesis, and real-time quantitative polymerase chain reaction (RT-qPCR)

Total RNA in tissue specimens and cultured cells was isolated using TRIzol™ Plus RNA Purification Kit (Thermo Fisher). Isolation of cytoplasmic and nuclear RNA from NSCLC cells was performed using the Cytoplasmic & Nuclear RNA Purification Kit (Norgen, Thorold, ON, Canada) in accordance with the manufacturer’s operating procedures. For RNase R digestion, total RNA derived from NSCLC cells was digested with 3 U/mg RNase R (Thermo Fisher). The reverse transcription reaction was performed using the HiScript III 1st Strand cDNA Synthesis Kit (Vazyme, Nanjing, China) or miRNA 1st Strand cDNA Synthesis Kit (Vazyme). After mixing AceQ SYBR qPCR Master Mix (Vazyme) with complementary DNA, qPCR was run on the Roche Applied Science LightCycler^TM^ 480 (Roche, Basel, Switzerland). Relative RNA levels of genes were calculated by the 2^−ΔΔCt^ method using U6 or GAPDH as the reference gene for normalization. Primer sequences are listed in Table 1.

**Table 1:** Primer sequences used for RT-qPCR

Genes	Primer sequences (5′–3′)
circ_0000735	Forward (F): 5′-GGCACTGCAGACCCATCTAT-3′
	Reverse (R): 5′-AGGCCCTTGAGTTTCACAGA-3′
P2RX1	F: 5′-CCTCATCAGCAGTGTCTCTGTG-3′
	R: 5′-CATGACCACGAAGGAGTTGTCC-3′
GAPDH	F: 5′-AGAAGGCTGGGGCTCATTTG-3′
	R: 5′-AGGGGCCATCCACAGTCTTC-3′
ITGB1	F: 5′-GGATTCTCCAGAAGGTGGTTTCG-3′
	R: 5′-TGCCACCAAGTTTCCCATCTCC-3′
miR-153-3p	F: 5′-CGCGTTGCATAGTCACAAAA-3′
	R: 5′-AGTGCAGGGTCCGAGGTATT-3′
U6	F: 5′-CTCGCTTCGGCAGCACA-3′
	R: 5′-AACGCTTCACGAATTTGCGT-3′

### 2.11 Dual-luciferase reporter assay

Fragments of wild-type circ_0000735 and 3′-UTR of ITGB1 mRNA containing the putative miR-153-3p binding sites and their mutant sequences were inserted into the psiCHECK-2 vector (Promega, Madison, WI, USA) to establish WT-circ_0000735, MUT-circ_0000735, WT-ITGB1 3′-UTR, and MUT-ITGB1 3′-UTR luciferase reporters, respectively. NSCLC cells were co-transfected with miR-153-3p mimic or miR-NC and a luciferase reporter vector. Two days later, the luciferase activities were measured using a dual-luciferase assay system (Promega).

### 2.12 *In vivo* experiments

The animal experiments were conducted with the approval of the Animal Ethics Committee of Hulunbeier Municipal People’s Hospital. Thirty-two BALB/c nude mice (Vital River Laboratory, Beijing, China) were randomly divided into four groups (eight mice in each group). Then, 16 mice in two groups were injected with A549 cells transduced with sh-NC and treated with intralipid or propofol. Analogously, 16 mice in two groups were injected with A549 cells transduced with sh-circ_0000735 and treated with intralipid or propofol. From Day 7, mice were treated with propofol (45 mg/kg) every 3 days via tail vein injection, and intralipid acted as vehicle control. Tumor volume was measured every 3 days (volume = (length × width^2^)/2) from propofol administration. Mice were maintained for 22 days before being sacrificed, following the appropriate protocols. Xenograft tumors were excised for subsequent analysis.

### 2.13 Immunohistochemistry (IHC)

Detection of ITGB1 protein in xenograft tumors was performed using IHC analysis as described previously [17]. Paraffin-embedded xenograft tissue sections were incubated with anti-ITGB1 (#ab134179, 1:100, Abcam), anti-Ki67 (#ab243878, 1:500, Abcam), or anti-MMP9 (#ab137867, 1:1,000, Abcam) antibodies.

### 2.14 Statistical analysis

All experiments were performed with a minimum of *n*  =  3 biological replicates and *n*  =  3 technical replicates. Results were represented as mean ± standard deviation using GraphPad Prism 8 (GraphPad Software, San Diego, CA, USA). Significance was assessed using Student’s *t*-tests (comparing data between two variances) or analysis of variance (comparing data with three or more variances) with Tukey’s *post hoc* test. Asterisks indicate significant differences between experimental groups (*P < 0.05, **P < 0.01, ***P < 0.001, and ****P < 0.0001).

## 3 Results

### 3.1 Propofol restrained proliferation, invasion, and induced apoptosis of NSCLC cells

**Figure 1:**
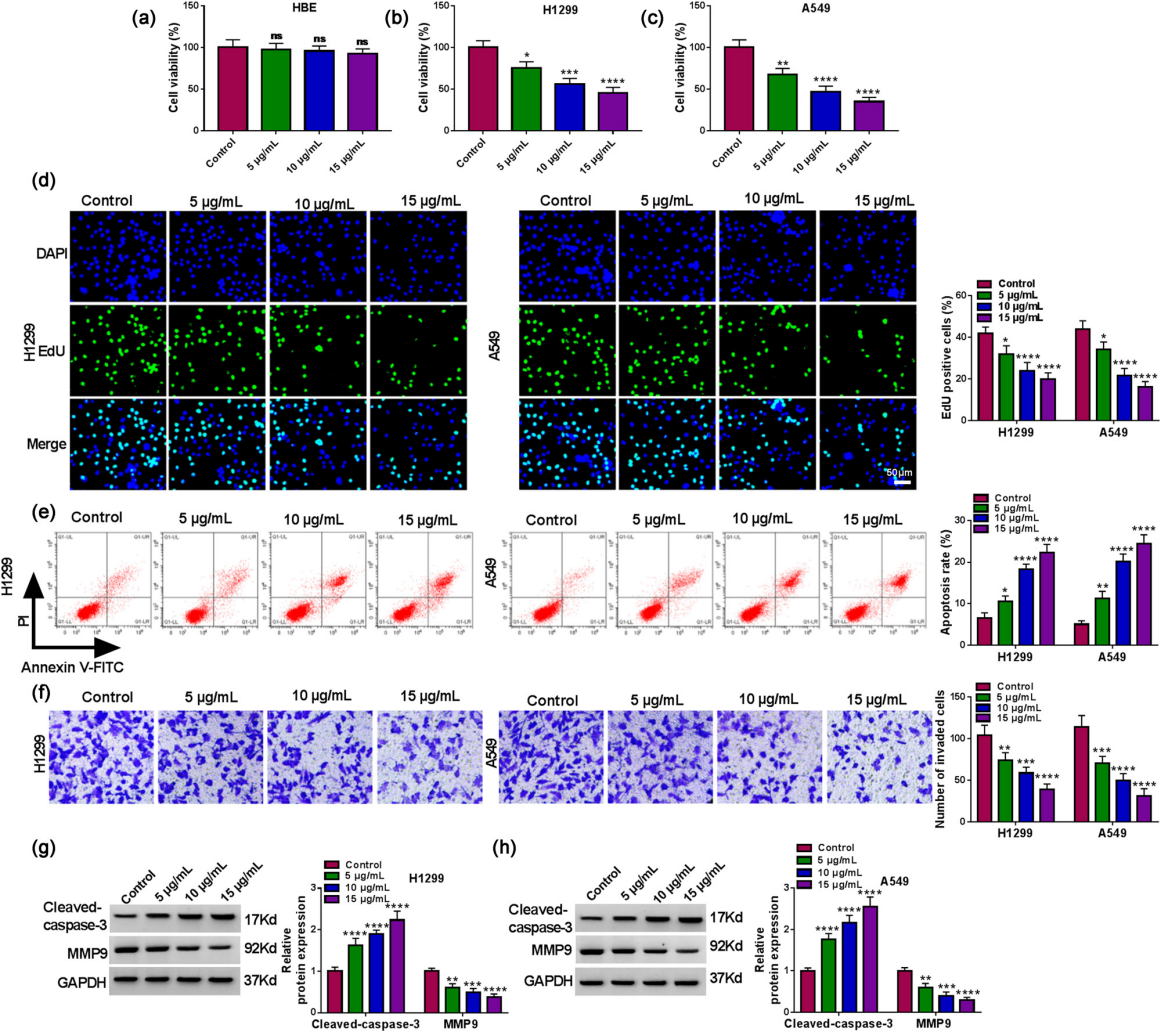
Propofol curbed cell proliferation, invasion, and induced cell apoptosis in NSCLC cells. (a) Cell viability was evaluated by MTT assays. The HBE cell line was subjected to 5, 10, and 15 μg/mL propofol for 48 h (*n* = 3). (b–f) Viability, proliferation, apoptosis, and invasion of NSCLC cells treated with 5, 10, and 15 μg/mL propofol were determined using MTT, EdU, flow cytometry, and transwell assays (*n* = 3). (g and h) Relative protein levels of Cleaved-caspase3 and MMP9 in NSCLC cells treated with 5, 10, and 15 μg/mL propofol were detected using western blotting (*n* = 3). *P < 0.01, **P < 0.01, ***P < 0.001, and ****P < 0.0001.

To evaluate the changes in NSCLC cell viability in response to anesthesia by propofol, we treated NSCLC cells with drug concentration gradients. Data in Figure 1a–c present that propofol restrained NSCLC cell viability in a concentration-dependent manner, but it did not affect the viability of HBE cells. Next, we assessed the impacts of propofol on NSCLC cell proliferation, apoptosis, and invasion. EdU assays exhibited that propofol significantly repressed NSCLC cell proliferation as its dose increases (Figure 1d). Flow cytometry assays showed that propofol led to a prominent increase in the proportion of apoptotic cells as its concentration increases (Figure 1e). Transwell invasion assays displayed that propofol resulted in a concentration-dependent suppression of NSCLC cell invasion (Figure 1f). We then assessed the protein levels of Cleaved-caspase3 and MMP9 in NSCLC cells in response to anesthesia by propofol. As shown in Figure 1g, propofol elevated the Cleaved-caspase3 protein level in NSCLC cells in a concentration-dependent manner. In contrast, propofol caused a marked decrease in the MMP9 protein level in a concentration-dependent manner (Figure 1h). Collectively, these results manifested that propofol restrained proliferation, invasion, and induced apoptosis of NSCLC cells as its concentration increases.

### 3.2 circ_0000735 was overexpressed in NSCLC

**Figure 2:**
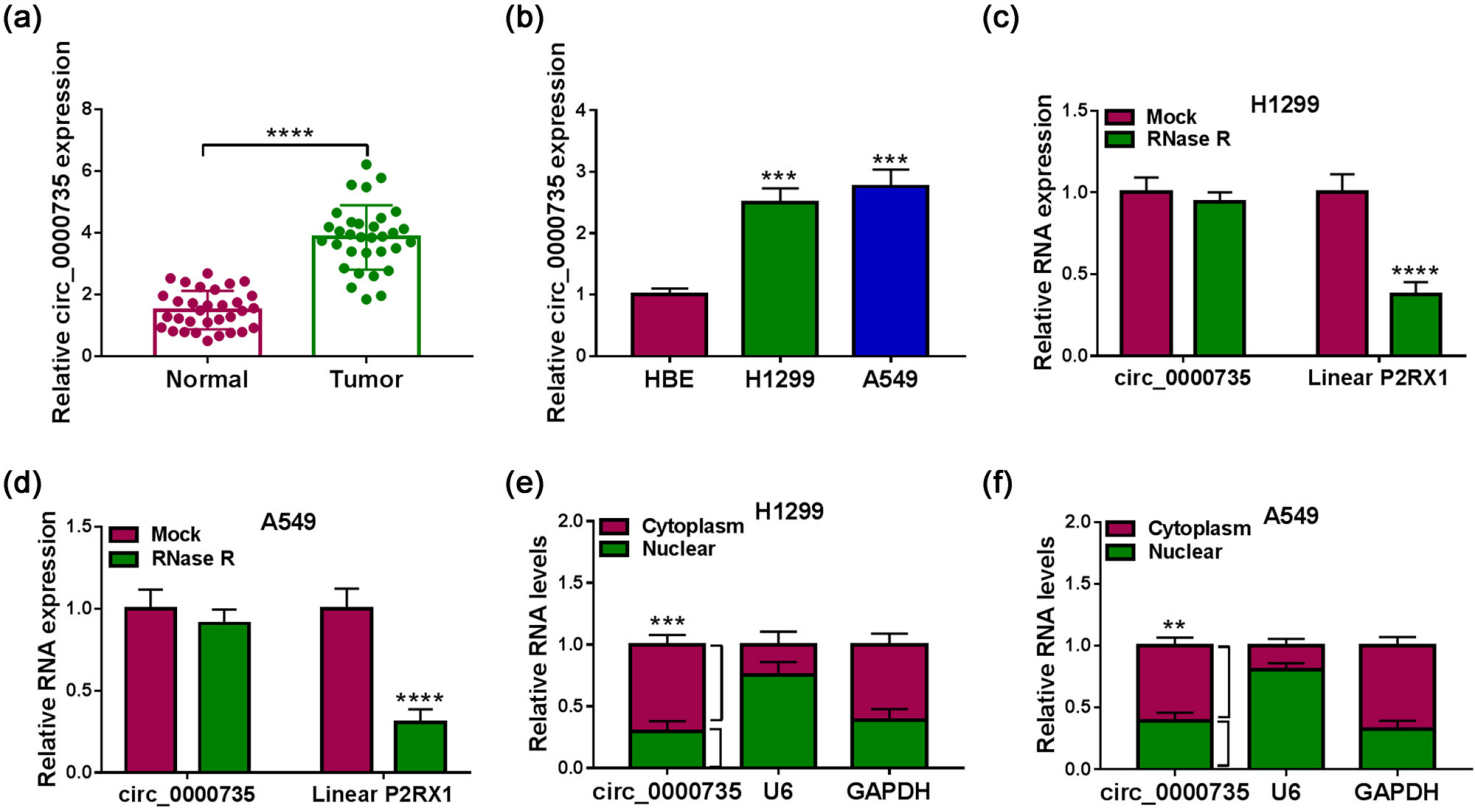
circ_0000735 was upregulated in NSCLC. (a and b) Relative expression of circ_0000735 in NSCLC tissues (*n* = 32) and cells (*n* = 3) was detected using RT-qPCR. (c and d) Relative expression of circ_0000735 in RNase R-digested NSCLC cell RNA was evaluated using RT-qPCR (*n* = 3). (e and f) After cell fractionation, the level of circ_0000735 in the nuclear and cytoplasmic fractions was analyzed using RT-qPCR (*n* = 3). U6 and GAPDH served as a positive control for nuclear and cytoplasmic fractions, respectively. ***P < 0.001 and ****P < 0.0001.

To validate the changes in circ_0000735 expression in NSCLC tissues and cells, we carried out RT-qPCR analysis with divergent primers. The results showed a particularly strong enhancement of circ_0000735 in NSCLC tissues and cells compared to their respective controls (Figure 2a and b). Resistance to the RNase R exonuclease was used to test the form of circ_0000735. We observed a prominent decrease in linear P2RX1 after RNase R treatment, but circ_0000735 was resistant to RNase R digestion, manifesting that circ_0000735 was more stable than its linear cognate mRNA (Figure 2c and d). A nuclear mass separation assay exhibited a dominantly cytoplasmic distribution of circ_0000735 (Figure 2e and f). Collectively, these results suggested that circ_0000735 expression was elevated in NSCLC.

### 3.3 Propofol controlled NSCLC cell proliferation, invasion, and apoptosis by regulating circ_0000735 expression

**Figure 3:**
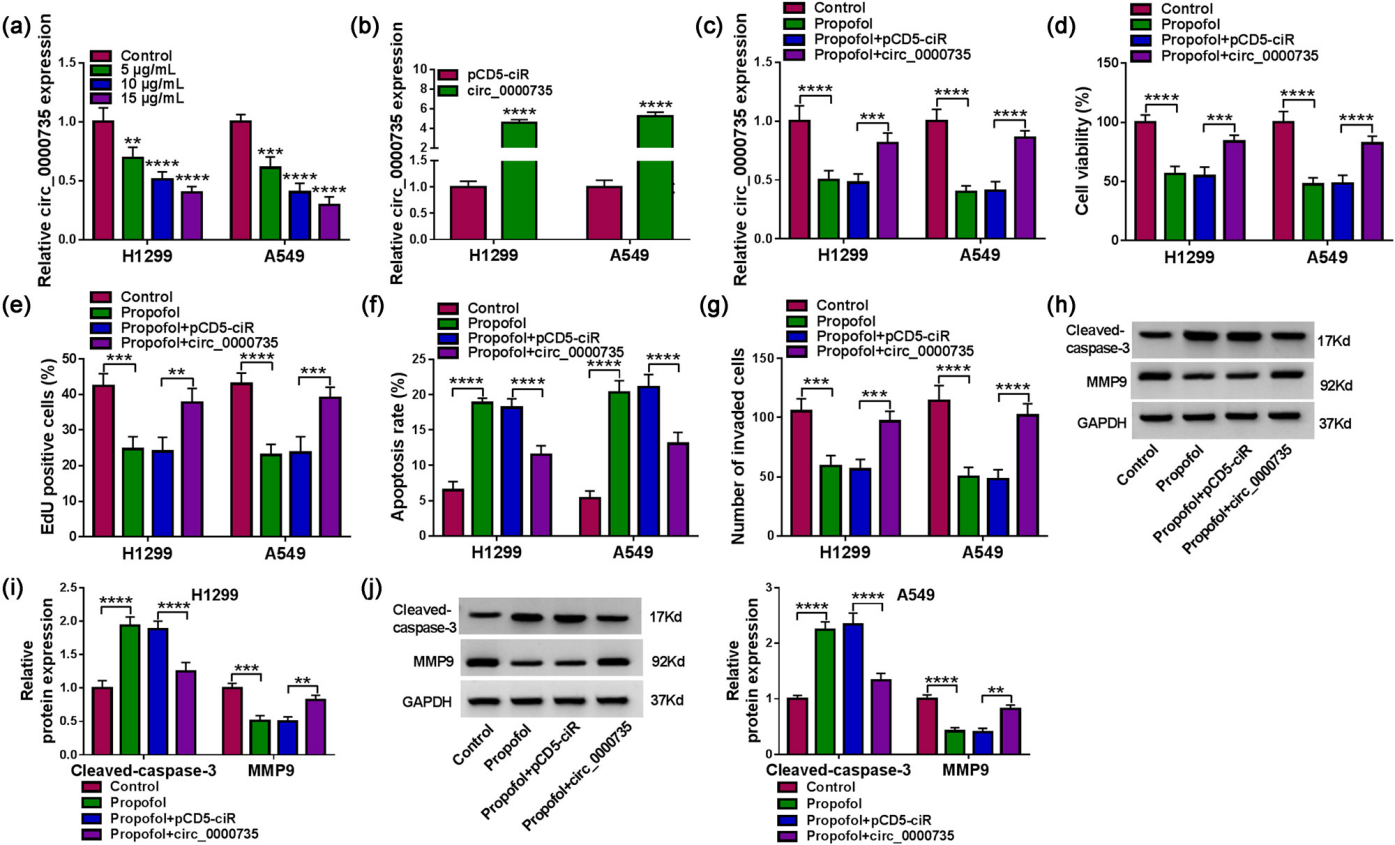
Propofol controlled cell proliferation, invasion, and apoptosis in NSCLC cells via regulating circ_0000735 expression. (a) Relative expression of circ_0000735 in NSCLC cells treated with 5, 10, and 15 μg/mL propofol was detected using RT-qPCR (*n* = 3). (b) Relative expression of circ_0000735 in NSCLC cells transfected with circ_0000735 or pCD5-ciR was evaluated using RT-qPCR (*n* = 3). (c) Influence of exogenous circ_0000735 on circ_0000735 expression in propofol-treated NSCLC cells was determined by RT-qPCR (*n* = 3). (d–g) Impacts of exogenous circ_0000735 on propofol-treated NSCLC cell viability, proliferation, apoptosis, and invasion were determined using MTT, EdU, flow cytometry, and transwell assays (*n* = 3). (h–j) Effects of exogenous circ_0000735 on protein levels of Cleaved-caspase3 and MMP9 in propofol-treated NSCLC cells were analyzed using western blotting (*n* = 3). **P < 0.01, ***P < 0.001, and ****P < 0.0001.

To verify the involvement of propofol and circ_0000735 in NSCLC cell proliferation, invasion, and apoptosis, we detected circ_0000735 expression in propofol-treated NSCLC cells. The results exhibited that propofol caused a distinct decrease in circ_0000735 expression, and 10 μg/mL of propofol was used for subsequent analysis (Figure 3a). Next, the circ_0000735 overexpression plasmid was constructed to explore the effects of circ_0000735 overexpression on proliferation, invasion, and apoptosis of propofol-treated NSCLC cells. After transfection, circ_0000735 was highly expressed in NSCLC cells (Figure 3b). Also, the downregulation of circ_0000735 in NSCLC cells caused by propofol was reversed after circ_0000735 introduction (Figure 3c). Also, exogenous circ_0000735 overturned propofol-mediated impacts on NSCLC cell proliferation, apoptosis, and invasion (Figure 3d–g). As expected, circ_0000735 overexpression reversed the elevation of Cleaved-caspase3 and the decrease of MMP9 in propofol-treated NSCLC cells (Figure 3h–j). Together, these results indicated that propofol curbed NSCLC cell proliferation, invasion, and induced NSCLC cell apoptosis via repressing circ_0000735 expression.

### 3.4 circ_0000735 was identified as a miR-153-3p decoy

**Figure 4:**
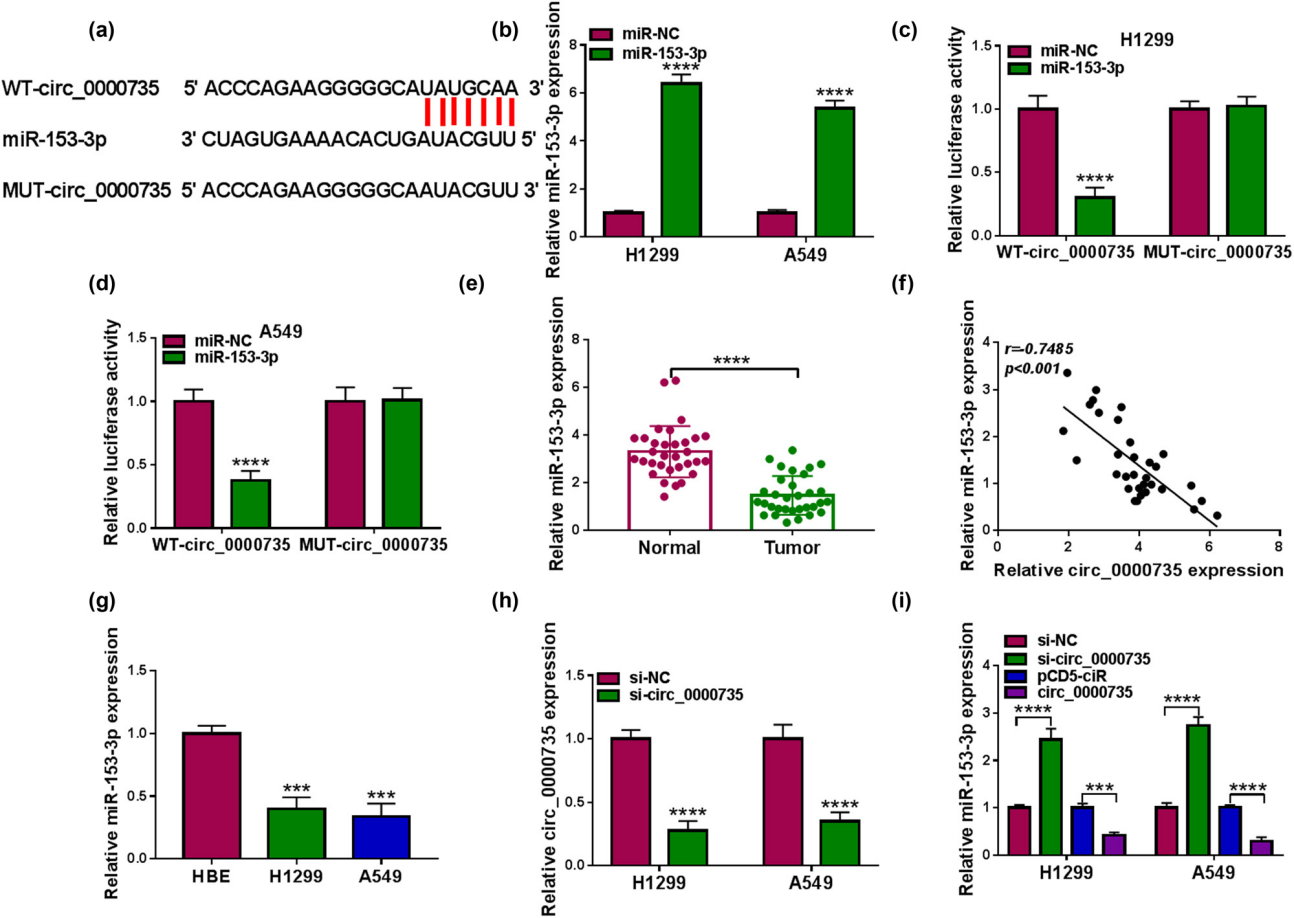
circ_0000735 served as a miR-153-3p decoy. (a) Representative sequence alignment of circ_0000735 with miR-153-3p. (b) RT-qPCR analysis of the transfection efficiency of miR-153-3p mimic (*n* = 3). (c and d) Analysis of the luciferase activities of the WT-circ_0000735 and MUT-circ_0000735 reporters in NSCLC cells with miR-153-3p mimic or miR-NC by dual-luciferase reporter assays (*n* = 3). (e) Evaluation of miR-153-3p in NSCLC tissues (*n* = 32) using RT-qPCR. (f) Correlation of circ_0000735 and miR-153-3p in NSCLC tissues was analyzed by Pearson’s correlation analysis. (g) Detection of miR-153-3p in NSCLC cells using RT-qPCR (*n* = 3). (h) Analysis of the interference efficiency of si-circ_0000735 using RT-qPCR (*n* = 3). (i) Impacts of circ_0000735 inhibition and overexpression on miR-153-3p expression were assessed using RT-qPCR (*n* = 3). ***P < 0.001 and ****P < 0.0001.

**Figure A1:**
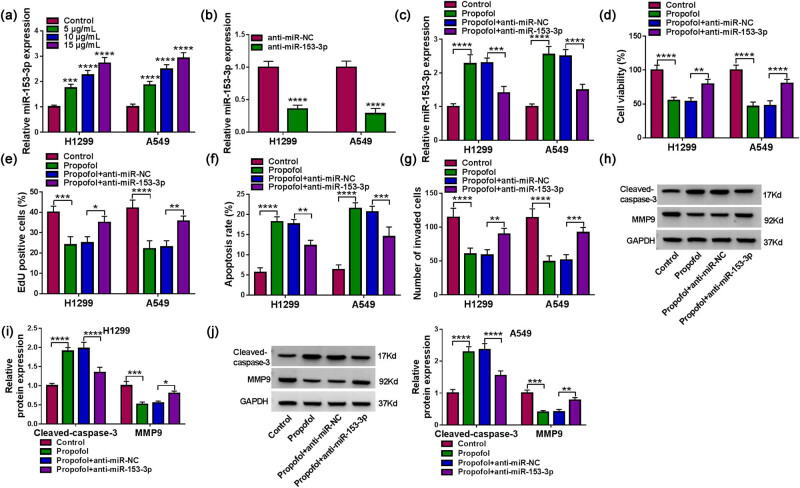
Overexpression of miR-153-3p lowered NSCLC cell malignancy. (a–d) The viability, proliferation, apoptosis, and invasion of NSCLC cells transfected with miR-NC or miR-153-3p were assessed by MTT, EdU, flow cytometry, and transwell assays (*n* = 3). ***P < 0.001 and ****P < 0.0001.

**Figure A2:**
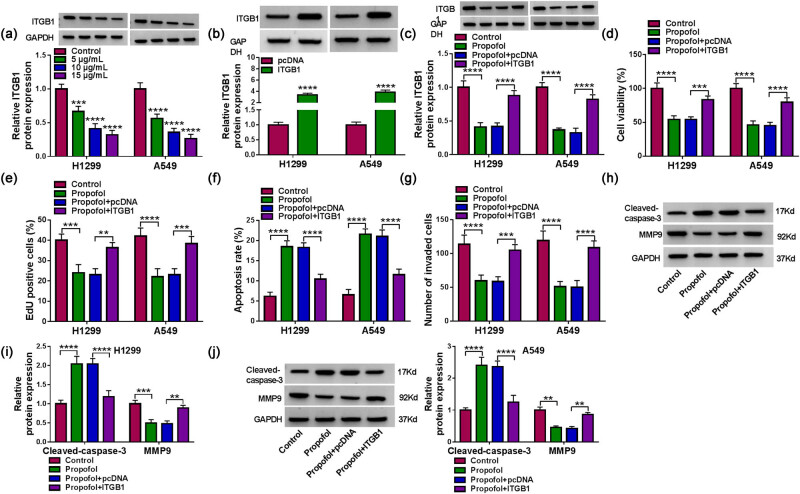
Propofol controlled NSCLC cell proliferation, invasion, and apoptosis by miR-153-3p. (a) Detection of miR-153-3p in NSCLC cells treated with 5, 10, and 15 μg/mL propofol using RT-qPCR (*n* = 3). (b) Assessment of the transfection efficiency of miR-153-3p inhibitor using RT-qPCR (*n* = 3). (c) Influence of miR-153-3p inhibitor on miR-153-3p expression in propofol-treated NSCLC cells was evaluated using RT-qPCR (*n* = 3). (d–g) Effects of miR-153-3p silencing on propofol-treated NSCLC cell viability, proliferation, apoptosis, and invasion were evaluated using MTT, EdU, flow cytometry, and transwell assays (*n* = 3). (h–j) Western blotting analysis of the impacts of miR-153-3p knockdown on protein levels of Cleaved-caspase3 and MMP9 in propofol-treated NSCLC cells (*n* = 3). *P < 0.01, **P < 0.01, ***P < 0.001, and ****P < 0.0001.

To further investigate the function of circ_0000735, we searched for miRs that might interact with circ_0000735. Through software prediction (Starbase2.0), we discovered that circ_0000735 had a complementary sequence to the miR-153-3p seed region (Figure 4a). To verify this hypothesis, NSCLC cells were overexpressed with miR-153-3p (Figure 4b). Dual-luciferase reporter assay revealed a marked decrease in the luciferase activity in NSCLC cells co-transfected with the WT-circ_0000735 reporter and miR-153-3p mimic, but there was no difference change in NSCLC cells co-transfected with the MUT-circ_0000735 reporter and miR-153-3p mimic (Figure 4c and d). As expected, miR-153-3p was lowly expressed in NSCLC tissues and it was negatively correlated with circ_0000735 expression (Figure 4e and f). Similar results were obtained with miR-153-3p in NSCLC cells (Figure 4g). MiR-153-3p overexpression decreased NSCLC cell viability, proliferation, elevated NSCLC cell apoptosis, and repressed NSCLC cell invasion (Figure A1a–d). Additionally, miR-153-3p was highly expressed in propofol-treated NSCLC cells as propofol dose increases (Figure A2a). The transfection efficiency of miR-153-3p inhibitor is presented in Figure A2b. Moreover, miR-153-3p inhibitor impaired the elevation of miR-153-3p in NSCLC cells mediated by propofol (Figure A2c). Also, miR-153-3p silencing reversed the impacts of propofol on NSCLC cell proliferation, apoptosis, and invasion (Figure A2d–g). And miR-153-3p knockdown overturned propofol-mediated effects on protein levels of Cleaved-caspase3 and MMP9 (Figure A2h–j). To assess the effect of circ_0000735 on miR-153-3p expression, we then designed a siRNA against circ_0000735. The interference efficiency of si-circ_0000735 is presented in Figure 4h. The exogenous circ_0000735 decreased miR-153-3p expression in NSCLC cells. In contrast, circ_0000735 silencing elevated miR-153-3p expression in NSCLC cells (Figure 4i). These results indicated that circ_0000735 acted as a miR-153-3p decoy.

### 3.5 ITGB1 was a target of miR-153-3p transfected

**Figure 5:**
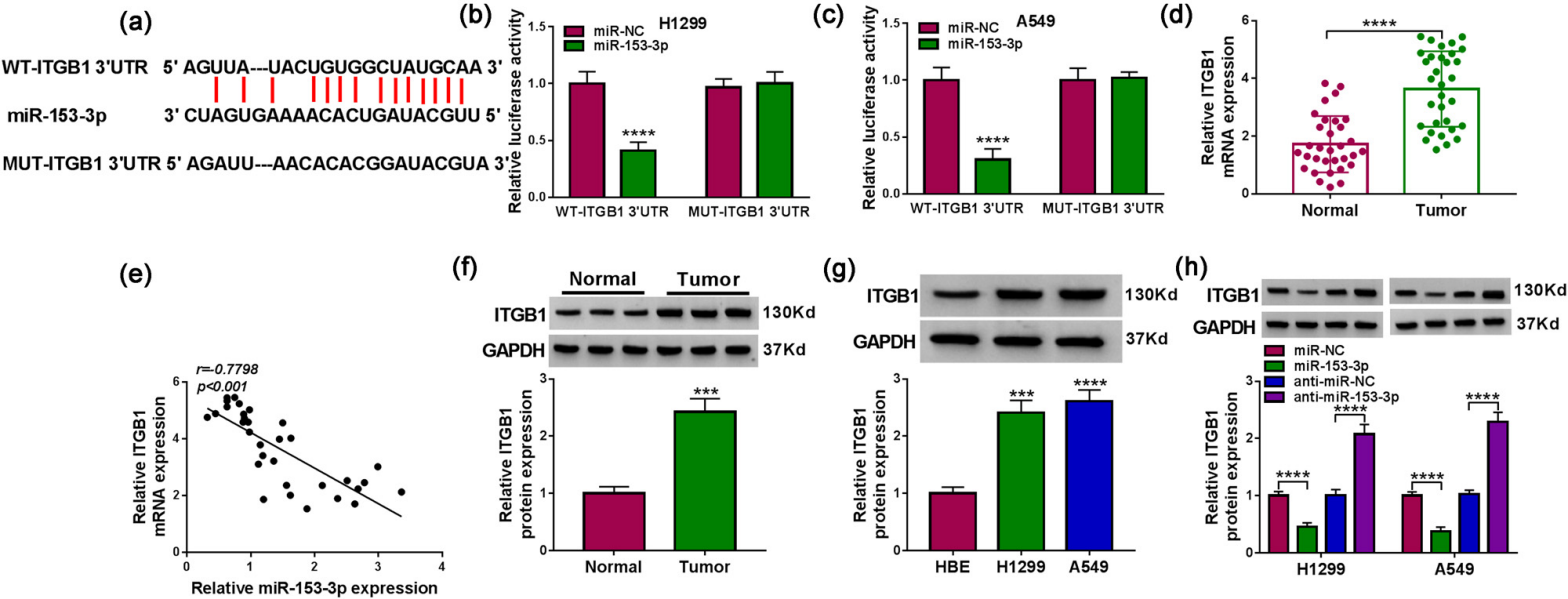
ITGB1 acted as a miR-153-3p target. (a) Sequence alignment of wild-type and mutant 3′-UTR of ITGB1 with miR-153-3p. (b and c) Luciferase activities of the WT-ITGB1 3′-UTR and MUT-ITGB1 3′-UTR reporters in NSCLC cells with miR-153-3p mimic or miR-NC were determined by dual-luciferase reporter assays (*n* = 3). (d) RT-qPCR analysis of ITGB1 mRNA in NSCLC tissues (*n* = 32). (e) Correlation of ITGB1 mRNA and miR-153-3p in NSCLC tissues was analyzed by Pearson’s correlation analysis. (f and g) Western blotting assessment of the ITGB1 protein level in NSCLC tissues (*n* = 32) and cells (*n* = 3). (h) Western blotting analysis of the effects of miR-153-3p inhibitor and mimic on the protein level of ITGB1 (*n* = 3). ***P < 0.001 and ****P < 0.0001.

**Figure A3:**
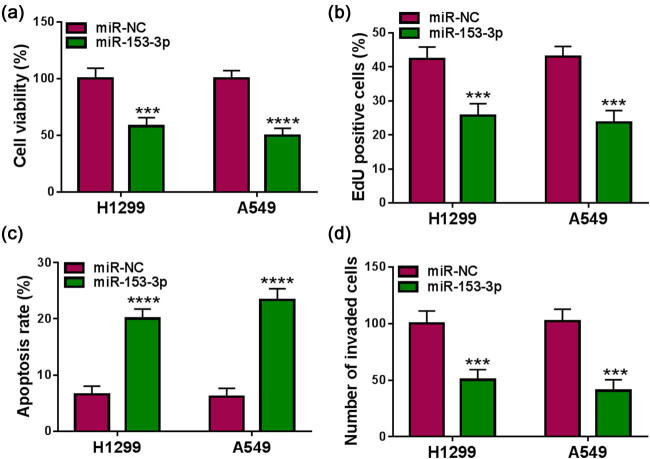
Propofol modulated NSCLC cell proliferation, invasion, and apoptosis by ITGB1. (a) Detection of ITGB1 protein in NSCLC cells treated with 5, 10, and 15 μg/mL propofol using western blotting (*n* = 3). (b) Assessment of the transfection efficiency of the ITGB1 overexpression plasmid using western blotting (*n* = 3). (c) Effect of ITGB1 overexpression on the protein level of ITGB1 in propofol-treated NSCLC cells was analyzed using western blotting (*n* = 3). (d–g) Effects of ITGB1 upregulation on propofol-treated NSCLC cell viability, proliferation, apoptosis, and invasion were determined using MTT, EdU, flow cytometry, and transwell assays (*n* = 3). (h–j) Western blotting evaluation of the effects of ITGB1 overexpression on protein levels of Cleaved-caspase3 and MMP9 in propofol-treated NSCLC cells (*n* = 3). **P < 0.01, ***P<0.001, and ****P < 0.0001.

We further explored the downstream mechanism of miR-153-3p. Based on bioinformatics analysis and preliminary experiments, ITGB1 was selected as a candidate gene. Sequence alignment of wild-type and mutant 3′-UTR of ITGB1 with miR-153-3p are shown in Figure 5a. Also, miR-153-3p mimic strongly decreased the luciferase activity of the WT-ITGB1 3′-UTR reporter but not the MUT-ITGB1 3′-UTR reporter (Figure 5b and c). Furthermore, the level of ITGB1 mRNA was upregulated in NSCLC tissues and it was negatively correlated with miR-153-3p (Figure 5d and e). Also, there was a prominent increase in the ITGB1 protein level in NSCLC tissues and cells (Figure 5f and g). In addition, propofol caused an obvious decrease in the ITGB1 protein level as its dose increases (Figure A3a). We then constructed the ITGB1 overexpression plasmid to explore the effects of ITGB1 overexpression on propofol-treated NSCLC cells. The transfection efficiency of the ITGB1 overexpression plasmid is shown in Figure A3b. Also, ectopic expression of ITGB1 rescued the downregulation of ITGB1 in propofol-treated NSCLC cells (Figure A3c). Moreover, upregulation of ITGB1 rescued propofol-mediated effects on NSCLC cell proliferation, apoptosis, and invasion (Figure A3d–g). In addition, the changes in the protein levels of Cleaved-caspase-3 and MMP9 in NSCLC cells mediated by propofol were overturned after ITGB1 overexpression (Figure A3h–j). As expected, miR-153-3p mimic repressed the ITGB1 protein level in NSCLC cells, while miR-153-3p inhibitor exerted an opposing effect (Figure 3h). Accordingly, these results indicated that ITGB1 acted as a miR-153-3p target.

### 3.6 circ_0000735 controlled NSCLC cell proliferation, apoptosis, and invasion via ITGB1

**Figure 6:**
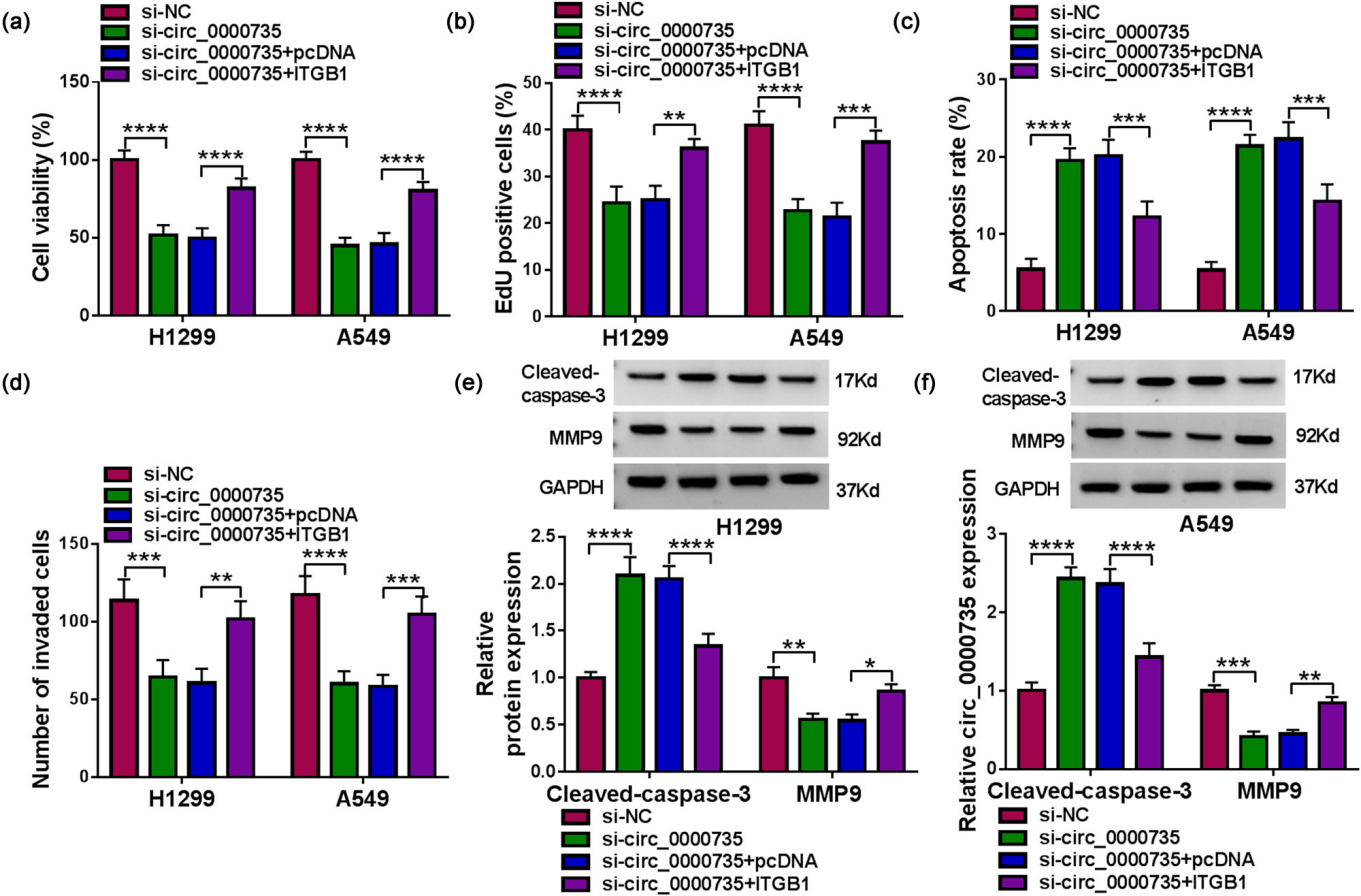
circ_0000735 regulated NSCLC cell proliferation, apoptosis, and invasion via ITGB1. (a–d) Effects of ITGB1 upregulation on si-circ_0000735-transfected NSCLC cell viability, proliferation, apoptosis, and invasion were determined using MTT, EdU, flow cytometry, and transwell assays (*n* = 3). (e and f) Western blotting analysis of the effects of ITGB1 overexpression on protein levels of Cleaved-caspase3 and MMP9 in si-circ_0000735-transfected NSCLC cells (*n* = 3). *P < 0.05, **P < 0.01, ***P < 0.001, and ****P < 0.0001.

We then conducted rescue experiments to identify whether circ_0000735 modulated NSCLC cell proliferation, apoptosis, and invasion via ITGB1. The results exhibited that transfection with si-circ_0000735 repressed cell viability, proliferation, and promoted cell apoptosis in NSCLC cells, but these effects caused by circ_0000735 knockdown were reversed by overexpression of ITGB1 (Figure 6a–c). Also, ectopic expression of ITGB1 overturned the repressive effect of circ_0000735 silencing on NSCLC cell invasion (Figure 6d). We also observed an overt increase in the Cleaved-caspase-3 protein level and a distinct reduction in the MMP9 protein level in si-circ_0000735-transfected NSCLC cells, but these trends were rescued by ITGB1 overexpression (Figure 6e and f). Collectively, these results manifested that circ_0000735 modulated NSCLC cell proliferation, apoptosis, and invasion via ITGB1.

### 3.7 Propofol modulated ITGB1 expression via the circ_0000735/miR-153-3p axis

**Figure 7:**
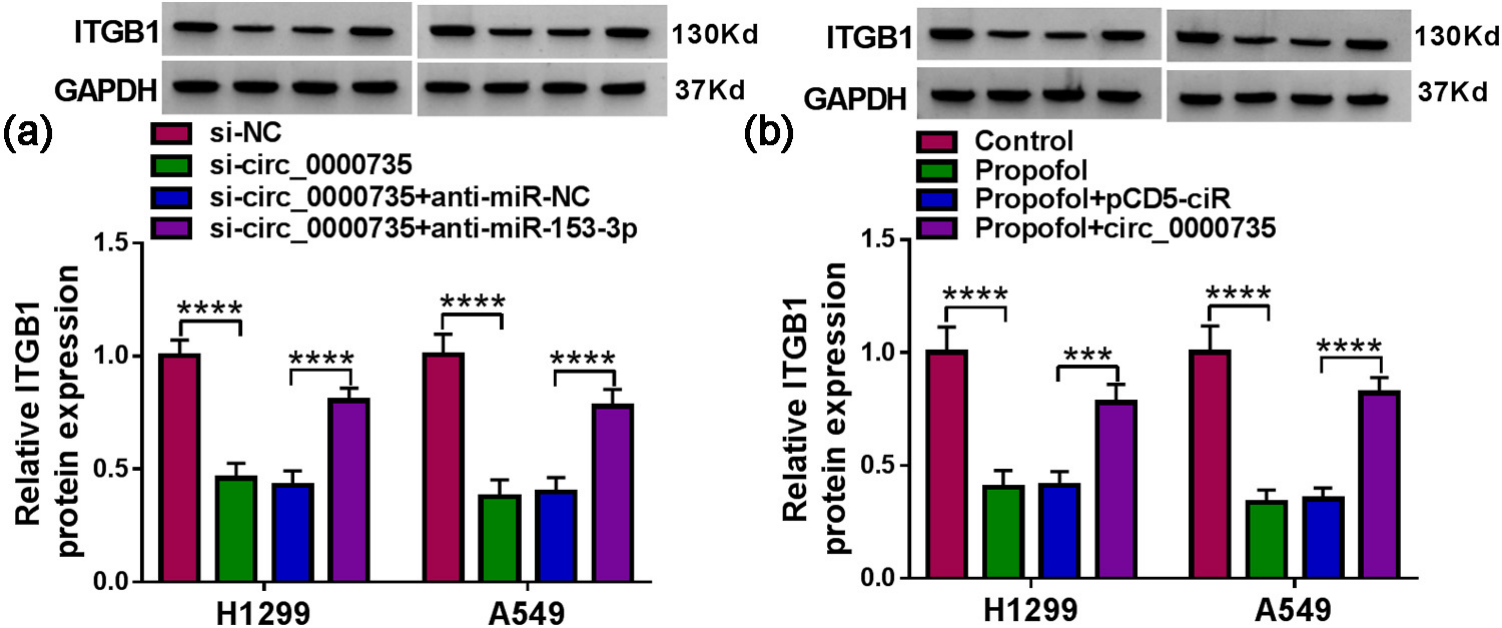
Propofol regulated ITGB1 expression via the circ_0000735/miR-153-3p axis. (a) Western blotting analysis of the effect of miR-153-3p inhibitor on the protein level of ITGB1 in si-circ_0000735-transfected NSCLC cells (*n* = 3). (b) Western blotting evaluation of the effect of circ_0000735 upregulation on the protein level of ITGB1 in propofol-treated NSCLC cells (*n* = 3). ***P < 0.001 and ****P < 0.0001.

Considering the above findings, we further explored whether circ_0000735 regulated ITGB1 expression via adsorbing miR-153-3p. Data in Figure 7a display that transection with miR-153-3p inhibitor rescued the downregulation of ITGB1 in NSCLC cells caused by circ_0000735 inhibition. Also, ectopic expression of circ_0000735 overturned the decrease in the ITGB1 protein level in NSCLC cells mediated by propofol (Figure 7b). Together, these results suggested that propofol regulated ITGB1 expression via the circ_0000735/miR-153-3p axis.

### 3.8 Propofol repressed A549 cell growth via downregulating circ_0000735 *in vivo*

**Figure 8:**
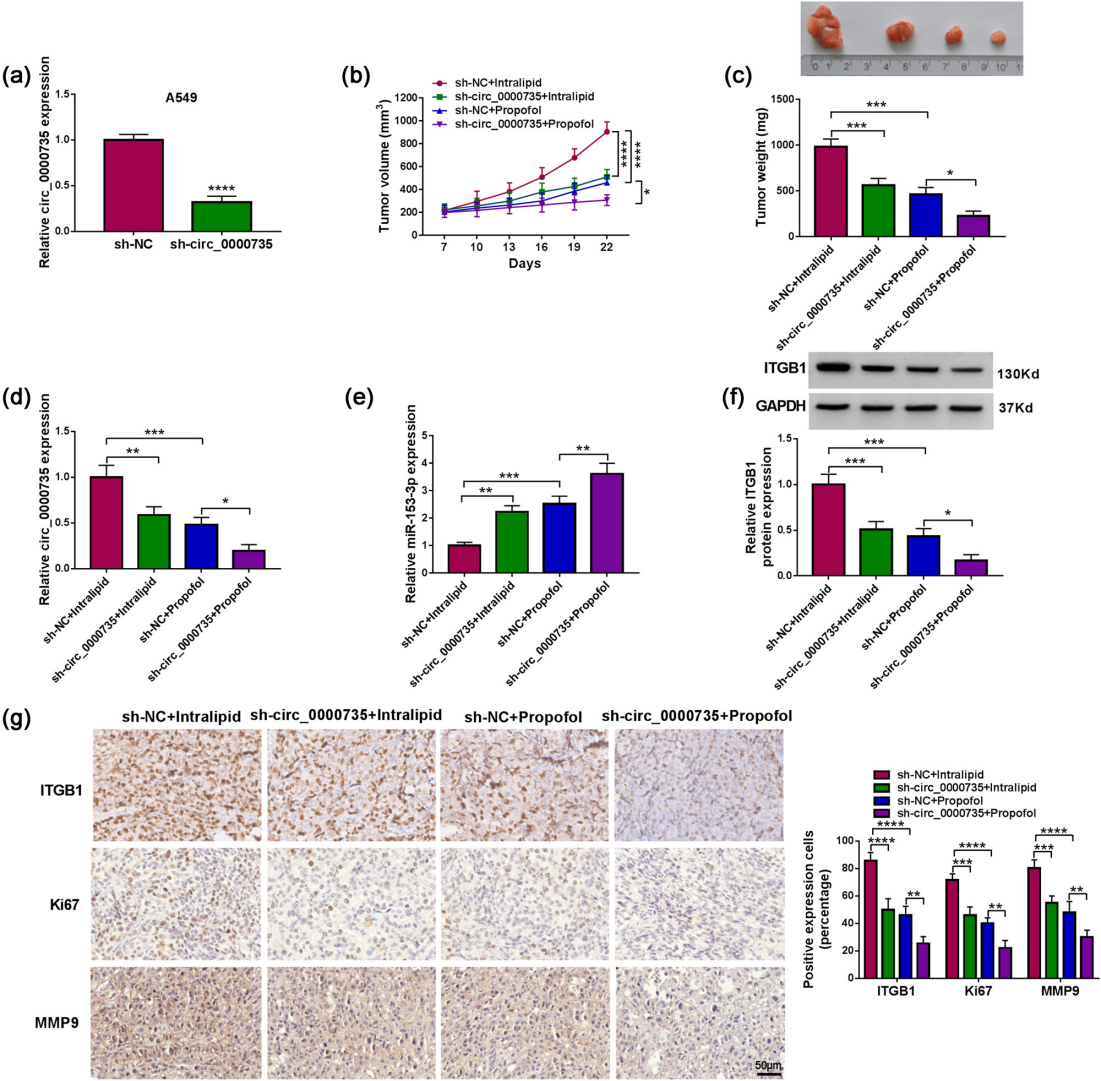
Propofol decreased xenograft tumor growth via downregulating circ_0000735 *in vivo*. (a) RT-qPCR analysis of circ_0000735 in A549 cells carrying sh-circ_0000735 or sh-NC (*n* = 3). (b and c) Growth curves, representative images, and weights of xenograft tumors in sh-NC + intralipid, sh-circ_0000735 + intralipid, sh-NC + propofol, and sh-circ_0000735 + propofol groups (*n* = 8). (d and e) Analysis of circ_0017639, miR-153-3p, and ITGB1 in xenograft tumors derived from sh-NC + intralipid, sh-circ_0000735 + intralipid, sh-NC + propofol, and sh-circ_0000735 + propofol groups by RT-qPCR or western blotting (*n* = 3). (g) Analysis of ITGB1, Ki67, or MMP9 in xenograft tumors derived from sh-NC + intralipid, sh-circ_0000735 + intralipid, sh-NC + propofol, and sh-circ_0000735 + propofol groups using IHC (*n* = 3). *P < 0.05, **P < 0.01, ***P < 0.001, and ****P < 0.0001.

To validate the above findings, we constructed xenograft tumor models. A549 cells stably expressing sh-circ_0000735 or sh-NC were constructed and the knockdown efficiency of sh-circ_0000735 is displayed in Figure 8a. The volume and weight of xenograft tumors derived from the sh-circ_0000735 + intralipid group were smaller and lighter than those from the sh-NC + intralipid group, indicating that circ_0000735 silencing decreased tumor growth *in vivo* (Figure 8b and c). We also observed that propofol treatment reduced the tumor volume and weight of mice in the sh-NC group, and the volume and weight of these tumors were further reduced after the silence of circ_0000735 (Figure 8b and c). Both propofol treatment and circ_0000735 silencing decreased circ_0000735 and ITGB1 levels while elevated miR-153-3p levels in xenograft tumors, but these trends mediated by propofol treatment were further strengthened after circ_0000735 silencing (Figure 8d–f). Consistently, both propofol treatment and circ_0000735 silencing reduced the number of ITGB1/Ki67/MMP9-positive cells in xenograft tumors, and the number of ITGB1/Ki67/MMP9-positive cells in the propofol treatment group was further reduced after circ_0000735 silencing (Figure 8g). Collectively, these results manifested that propofol repressed NSCLC cell growth via downregulating circ_0000735 *in vivo*.

## 4 Discussion

Recently, the anti-tumor activity of propofol in cancer has garnered significant interest [18]. Some researchers have revealed that propofol restrained cell malignant phenotypes via downregulating FOXM1 through regulating the circ_RHOT1/miR-326 axis in NSCLC [19] and the circ_TADA2A/miR-155-3p axis [12] or circ_ERBB2/miR-7-5p axis [13] in lung cancer. Our work uncovered that propofol repressed NSCLC cell malignant phenotypes through downregulating ITGB1 by regulation of the circ_0000735/miR-153-3p axis, at least in part.

Previous studies have demonstrated that circ_0000735 exerts an oncogenic role in diverse cancers. Zheng et al. manifested that circ_0000735 facilitated cell invasion and proliferation via sequestration of miR-502-5p [20]. Also, upregulated circ_0000735 decreased cell sensitivity to docetaxel via functioning as a miR-7 sponge in prostate cancer [21]. Furthermore, high circ_0000735 expression could forecast the severity of NSCLC, and circ_0000735 upregulation promoted NSCLC cell malignant phenotypes via sponging miR-1182 and miR-1179 [22]. Also, circ_0000735 elevated BMPER or FAM83F expression via adsorbing miR-940 or miR-635, respectively, thus facilitating NSCLC progression [16,23]. Consistent with the previous studies [16,22,23], circ_0000735 was overexpressed in NSCLC in our study. Moreover, propofol caused a decrease in circ_0000735 expression in NSCLC cells. Upregulation of circ_0000735 reversed the suppressive impacts of propofol on NSCLC cell viability, proliferation, invasion, and the promoting influence of propofol on NSCLC cell apoptosis. Also, propofol decreased xenograft tumor growth, and circ_0000735 expression was lower in xenograft tumors derived from mice treated with propofol. Hence, we inferred that propofol curbed NSCLC cell malignant phenotypes by downregulating circ_0000735.

Through bioinformatics analysis and dual-luciferase reporter assays, circ_0000735 was identified as a miR-153-3p sponge. It was reported that miR-153-3p played an anti-tumor activity in various cancers, such as medullary thyroid cancer [24], cervical cancer [25], and oral squamous cell cancer [26]. Also, miR-153-3p upregulation mediated by NEAT1 knockdown suppressed NSCLC cell malignant phenotypes [27]. Moreover, miR-153-3p repressed NSCLC growth and stem cell-like phenotype by targeting Jagged1 [28]. Here, propofol resulted in an increase in miR-153-3p expression in NSCLC cells, and miR-153-3p silencing reversed propofol-mediated effects on NSCLC cell viability, proliferation, invasion, and apoptosis. These results manifested that propofol might restrain NSCLC cell malignant phenotypes by regulating the circ_0000735/miR-153-3p axis.

The integrin family includes a group of heterodimeric cell surface transmembrane proteins that can mediate cell–cell and cell–matrix interactions [29]. ITGB1, a subunit of the integrin family, is aberrantly overexpressed in solid tumors and is related to the poor prognosis of diverse cancers [30]. A series of studies have revealed that ITGB1 acted as an oncogene in NSCLC [31–33]. The downregulation of ITGB1 mediated by miR-374b [34], miR-134 [35], and miR-384 [36] repressed NSCLC cell malignant phenotypes. Here, ITGB1 served as a miR-153-3p target, and propofol led to a decrease in the ITGB1 protein level. Moreover, ITGB1 upregulation offset propofol-mediated impacts on NSCLC cell viability, proliferation, invasion, and apoptosis. Importantly, propofol regulated ITGB1 expression by regulation of the circ_0000735/miR-153-3p axis. Thus, we inferred that propofol regulated NSCLC progression through regulation of the circ_0000735/miR-153-3p/ITGB1 axis.

All in all, propofol restrained NSCLC progression through downregulating circ_0000735, which regulated ITGB1 expression through functioning as a decoy of miR-153-3p. The research validates the inhibiting effect of propofol on NSCLC cell growth and offers a novel mechanism by which propofol represses NSCLC progression by the circ_0000735/miR-153-3p/ITGB1 axis.
